# The Effect of Arch Height and Material Hardness of Personalized Insole on Correction and Tissues of Flatfoot

**DOI:** 10.1155/2017/8614341

**Published:** 2017-06-12

**Authors:** Shonglun Su, Zhongjun Mo, Junchao Guo, Yubo Fan

**Affiliations:** ^1^Key Laboratory for Biomechanics and Mechanobiology of Ministry of Education, School of Biological Science and Medical Engineering, Beihang University, Beijing 100086, China; ^2^Beijing Key Laboratory of Rehabilitation Technical Aids for Old-Age Disability, Key Laboratory of Rehabilitation Technical Aids Analysis and Identification of the Ministry of Civil Affairs, National Research Centre for Rehabilitation Technical Aids, Beijing 100176, China

## Abstract

Flat foot is one of the common deformities in the youth population, seriously affecting the weight supporting and daily exercising. However, there is lacking of quantitative data relative to material selection and shape design of the personalized orthopedic insole. This study was to evaluate the biomechanical effects of material hardness and support height of personalized orthopedic insole on foot tissues, by in vivo experiment and finite element modeling. The correction of arch height increased with material hardness and support height. The peak plantar pressure increased with the material hardness, and these values by wearing insoles of 40° were apparently higher than the bare feet condition. Harder insole material results in higher stress in the joint and ligament stress than softer material. In the calcaneocuboid joint, the stress increased with the arch height of insoles. The material hardness did not apparently affect the stress in the ankle joints, but the support heights of insole did. In general, insole material and support design are positively affecting the correction of orthopedic insole, but negatively resulting in unreasonable stress on the stress in the joint and ligaments. There should be an integration of improving correction and reducing stress in foot tissues.

## 1. Introduction

The foot and ankle is a complex structure with stability and elasticity, consisting of 28 bones, more than 30 joints, and many intertwined ligaments and tendons [1]. It plays an important role in supporting the body weight. Foot deformity is a very common disease, not only causing pain but also seriously affecting people's health and daily activities [2]. Flatfoot is one of the common deformities, which has a high incidence rate in the Chinese youth population. During the growth period, the mild and moderate flattened feet will be corrected with the growth of soft tissue and bones. Severe flatfoot may result in ligament relaxation, muscle weakness, joint distortion, limb pain, ankle injury, ulcer, and other clinical symptoms, which need for conservative correction or surgical intervention [3, 4].

Clinically, correction of orthopedic insoles combined with manual reposition is one of the primary conservation therapies for flexible flatfoot of young patient [3, 4]. Orthopedic insoles have been revealed to be an effective treatment for flatfoot by elevating the arch height and recovering the body weight supporting and force transmission [5]. Due to the difference between individuals (soft tissue material properties, flatness, etc.), customized orthopedic insole needs to be produced. In the customization process, the insole shape design and material selection of the insole seriously depended on the experience of pedorthist, lack of quantitative theoretical support. Previous study showed that the custom-molded insole could reduce stress compared with the flat insole. The thickness, heel's height, and materials of proper insole could minimize the peak plantar stress and achieve uniform stress distribution [6]. The material and arch height of insole in the sport shoes have a significant effect on biomechanical characteristics of the foot [7, 8]. Therefore, it is necessary to research the biomechanical effect of the design parameters of orthopedic insole, including the material and shape of support and the correction effect of foot arch height, and explore the influence on the foot tissues. Quantified assessment of the design parameters is a benefit to the future design of orthopedic insole to improve treatment effect and to reduce the adverse effects on the patient's foot tissues [9–12].

This study was to evaluate the effects of design parameters including material hardness and support arch height of personalized orthopedic insole on the correction of foot arch and plantar pressure distribution by in vivo experiments and to evaluate the biomechanical effects on the foot tissues, including stress distribution on joint cartilage and ligaments using finite element modeling.

## 2. Material and Methods

Based on a specific patient with flatfoot, this study was to evaluate the effect of design parameters including material hardness (Shore A, 30°, 35°, and 40°) and support arch height (27 mm, 30 mm, and 33 mm) of orthopedic insole on the correction effect of foot arch and plantar pressure distribution by in vivo experiment. In the second step, finite element modeling was adopted to evaluate the biomechanical effects on the foot tissues, including stress distribution on joint cartilage and ligaments.

### 2.1. Subject and Data Processing

A young subject (12 years old, 160 cm height, and 55 kg weight) with severe flatfoot participated this study. The participant was explained on the research purpose and signed the consent form. At first, a series of customized insole of the subject were achieved for the in vivo experiment. CT images including the whole foot and portion crus of the subject were obtained at 0.5 mm interval and 0.6 mm resolution using a CT scanner (Brilliance iCT, Philips, Netherlands) under a quasi-weight-bearing condition with bare feet, used in the geometrical modeling. The quasi-weight-bearing status (275 N per foot) was produced by a customized equipment that consisted of an adjustable frame and a plane pressure measuring system (Pedar, Novel, Germany). The images were also checked to ensure that the flatfoot does not exhibit any radiographic evidence of tissue deformity symptoms. The study plan was approved by the Ethical Committee of the corresponding institute.

### 2.2. Customized Insole of the Subject

**Figure 1:**
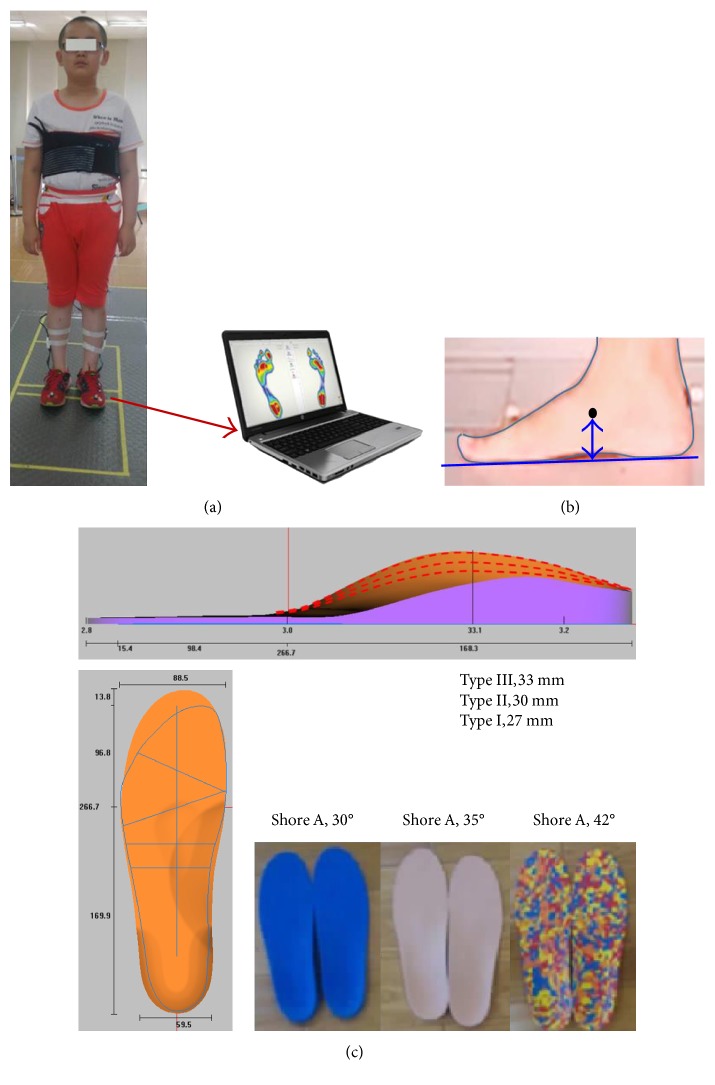
Illustration of in vivo measurement, (a) plantar pressure measurement, (b) displacement measurement of the navicular bone, and (c) insole material hardness and design sketch.

The foot profile of the subject under weight-bearing and non-weight-bearing conditions was obtained as point contours using a 3D foot scanner (Infoot, I-Ware Laboratory Co. Ltd. JPN). An insole design software (GeBioM, Go_Tec Inc. GER) was used to develop the 3D geometrical model of customize insoles according to the participant's foot contours. The general thickness of the insole was designed as 7 mm. In clinical practice, the orthopedic insole is produced basing on non-weight-bearing condition, in which the arch shape was close to the normal configuration. Since the arch height of the subject is about 23 mm in the non-weight-bearing condition, the initial total arch height was setting at 30 mm and ±3 mm that were chosen for this parametric study and that were 27 mm (type I), 30 mm (type II), and 33 mm (type III) in sequence, as shown in Figure 1. All these settings were guided by a pedorthist and a therapist. Three kinds of materials, with different hardness (Shores A, 30°, 35°, and 40°), were chosen for its high popularity and sustainability for orthopedic insoles.

### 2.3. In Vivo Measurement

Wireless in-foot pressure measurement system (F-Scan, Novel Inc., US), which consists of 100 capacitive sensors, was used to test the plantar pressure by wearing the nine different orthopedic insoles (3 arch heights, 3 materials), as shown in Figure 1(a). In the meanwhile, the distance between the mark point on the navicular bone and ground was measured to evaluate the correction effect of different insoles, as shown in Figure 1(b), and to be used in the next FE modeling as the boundary loading conditions to explore the effect on the foot tissues.

### 2.4. Finite Element Modeling of Flatfoot

**Figure 2:**
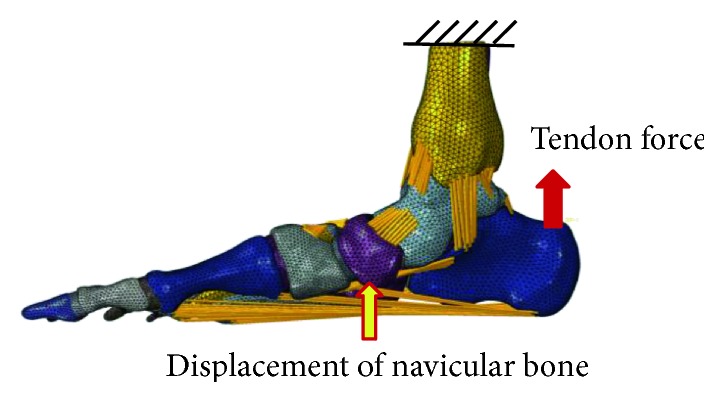
Finite element model of flatfoot and its components.

Medical image processing software (Mimics 10.1, Materialise Inc., Belgium) was used to segment the CT images to acquire the boundaries of each foot bone and skin surface to reconstruct the geometry models. Then, the geometries were imported into a reverse engineering software (Rapidform XOR3, INS Techology Inc., US) to edit the geometry with operations, such as smoothing and partition, and reconstruct the geometry as nurbs format. In total, the model included 28 bony structures, including tibia, fibia, talus, and calcaneus, as shown in Figure 2. The joint regions on the bone were extracted separately and rebuilt into solid blocks by thickening operation with a depth of 0.4 mm to reconstruct joint cartilages. Seventy-two major ligaments, deep fascia, superficial fascia, and nine major extrinsic muscle groups controlling foot movement were included and defined by connecting the corresponding anatomical attachment sites on the bones.

The FE package, ABAQUS 6.13 (Simulia Inc., US), was used for assembling the foot components, creating of the FE mesh, and implicit solver was employed for the subsequent analysis. The insertion points of the ligaments, the interface of skin, and the interfaces of the joint cartilage were fixed onto the corresponding regions on the bony structure by tie constraint formulation to assemble the foot components. The interaction among interfaces of joint cartilage was assigned with frictionless sliding contact formulation.

The material properties of each component of the foot tissues and insoles were selected from the literature and listed in Table 1 [13–18]. The element type of the foot tissues and insoles obtained by multimeshing techniques is listed in Table 1, together with the material properties. The element sizes for bone and skin were 2.5 and 1.5 mm, respectively, that resulted in a total of 94,522 nodes and 306,289 elements. Convergence within 3% in joint cartilage stress was achieved in bare feet weight-bearing condition, to ensure that the results were irrelevant to the mesh density [19, 20].

Prior to applying the correct loading on the navicular bone condition, the superior cross section surface of the tibia, fibula, and skin was fixed at the six degrees of freedom, and the initial tendon force of 375 N was properly adjusted until the foot was properly aligned relative to the insole [15–17]. In the second step, the displacement of the navicular bone achieved in the previously described in vivo experiment was applied on the reference point of the navicular bone in vertical direction by keeping the freedom in other direction to allow coupling motion and to simulate the correcting effect of different orthopedic insoles.

### 2.5. Finite Element Model Validation

Validation is one of the important stages in finite element modeling. This flatfoot model was validated by comparing the plantar pressure in FE model and in vivo measurement, under weight-bearing with bare foot on a flat plane.

## 3. Results

Nine customize insoles (3 arch heights, 3 hardness materials) were manufactured. The height between the navicular bone and the ground and the plantar pressure by wearing the different customize insoles were achieved in the in vivo experiment. By the finite element modeling, the stress in the joint cartilage and ligaments was extracted.

### 3.1. Validation of Flatfoot Model

**Figure 3:**
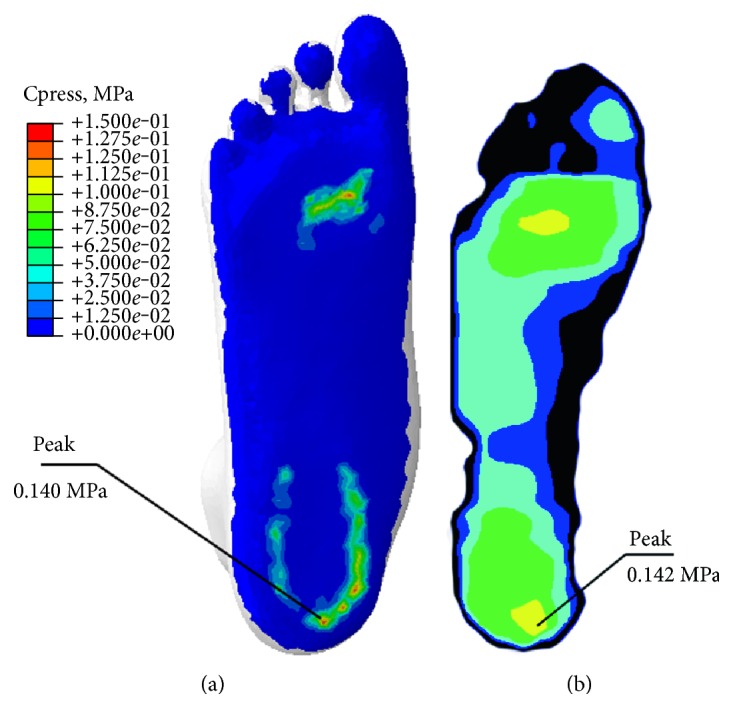
Plantar pressure distribution in (a) FE model and (b) F-Scan measurement.

As shown in Figure 3, the plantar pressure distribution in FE model was similar with F-Scan data and the peak plantar pressure was 140.0 KPa and 142 KPa in FE model and F-Scan measurement, respectively. It meant that the FE flatfoot could provide reasonable results in the present research purpose.

### 3.2. Correction in Foot Arch Height

**Figure 4:**
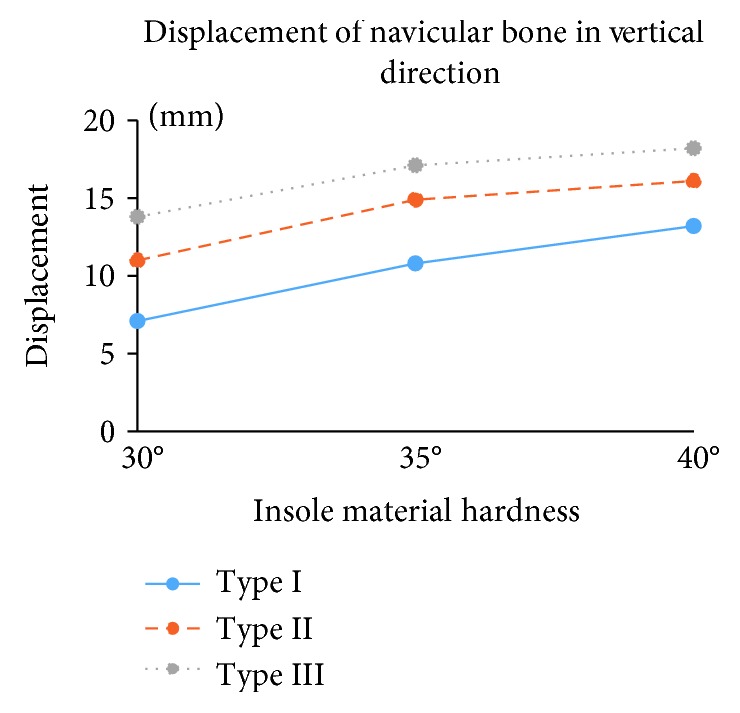
Displacement of the navicular bone in the vertical direction.

In bare foot and weight-bearing condition, the distance between the navicular bone and the foot bottom (arch height) was about 16.6 mm. The displacement of the navicular bone in the vertical direction by wearing orthopedic insoles is shown in Figure 4. The foot arch height increased with the material hardness and arch shape of the insole. By wearing insoles made up by material with hardness of 30°, the arch height increased by 43%, 66%, and 83% with insoles of types I, II, and III, respectively. By wearing the insoles with material hardness of 35°, the arch height increased by 65%, 90%, and 103% with insoles of types I, II, and III, respectively. By wearing insoles with material hardness of 40°, the arch height increased by 80%, 97%, and 110% with insole of types I, II, and III, respectively.

### 3.3. Plantar Pressure Distribution

**Figure 5:**
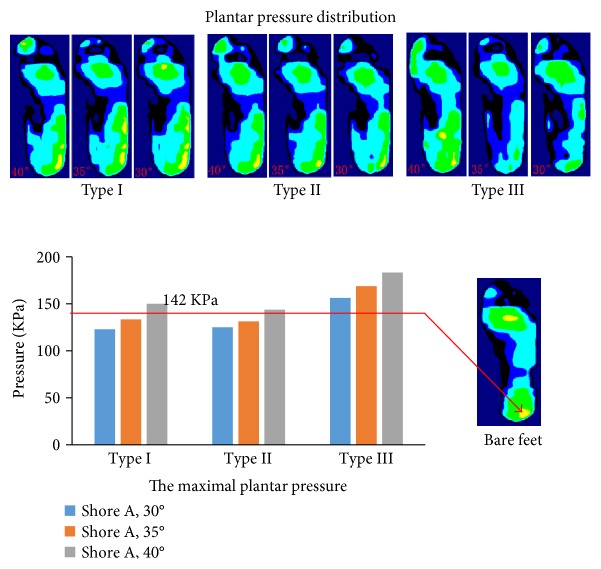
The maximal plantar pressures and pressure distribution in the in vivo measurement.

Plantar pressure is one of the most important parameters reflecting the interaction between the foot and insole. The peak plantar pressure and its distribution in the in vivo experiment are shown in Figure 5. In bare feet condition, the maximal plantar pressure was about 142 KPa. Regardless the insole shape, the peak plantar pressure increased with the material hardness. Regardless the material hardness, the maximal plantar pressure was not apparently increased by wearing types I and II insoles. However, the pressure was apparently increased by wearing type III insoles. By wearing insoles made up by the material hardness of 40°, regardless the shapes (types I, II, and III), the plantar pressure was higher than the bare feet condition.

### 3.4. Stress in the Primary Foot Joints

**Figure 6:**
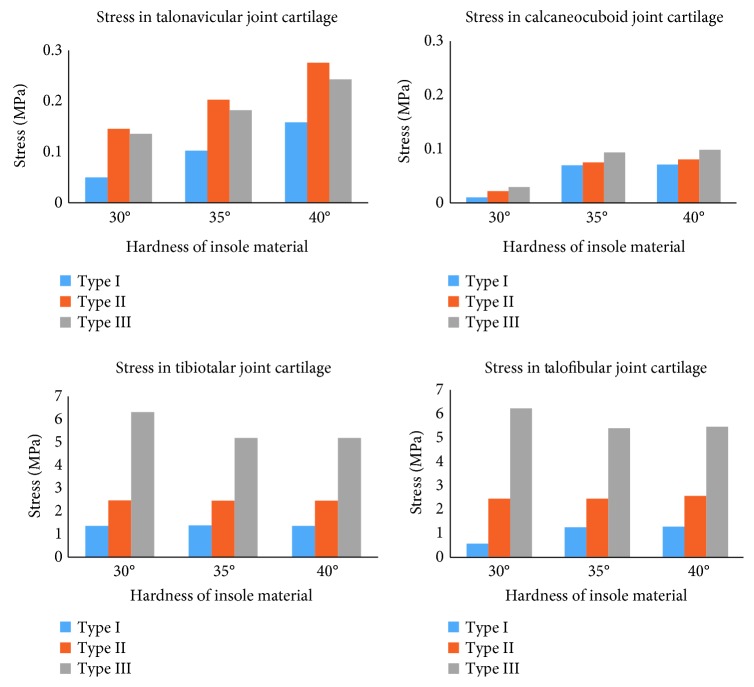
The maximal stress in joint cartilage in the finite element model.

The maximal stress in the cartilage of the primary foot joints (talonavicular, calcaneocuboid, tibiotalar, and talofibular joints) is shown in Figure 6. In the joint of the middle foot (talonavicular joint and calcaneocuboid joint), the maximal stress increased with the material hardness. In the calcaneocuboid joint, the stress increased with the arch height of insoles. However, in the talonavicular joint, type II insole results in the greatest cartilage stress. The stress in the middle joint was relatively much lower than the ankle joints (tibiotalar joint and talofibular joint). The material hardness did not apparently affect the stress in the ankle joints, but the arch height of orthopedic insoles did.

### 3.5. Stress in the Primary Foot Ligaments

**Figure 7:**
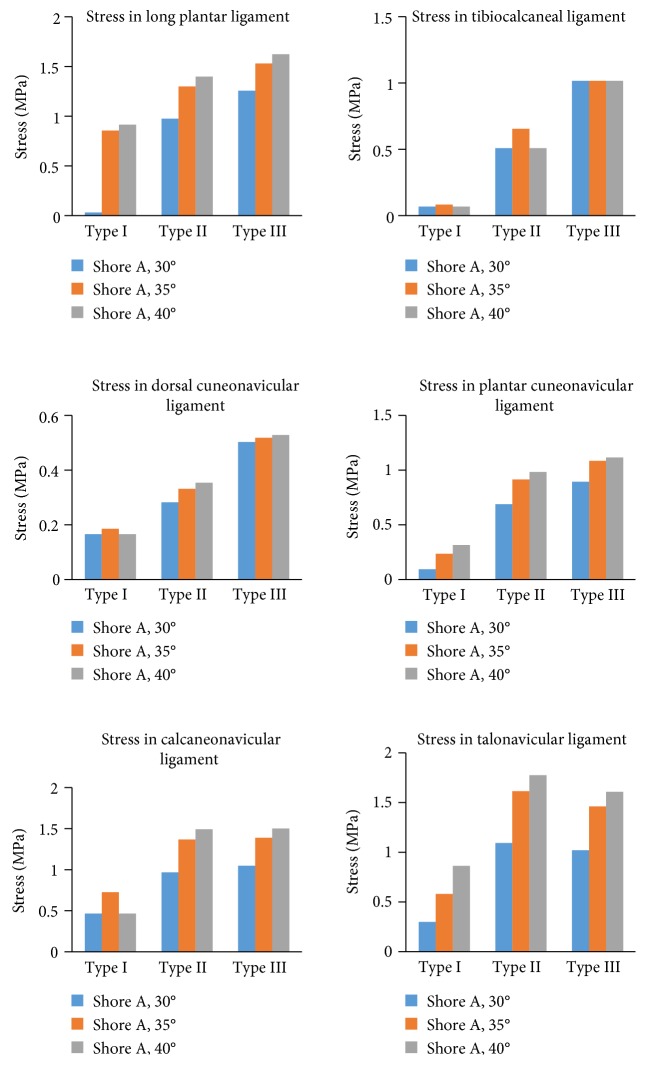
The maximal stress in the foot ligaments in the finite element model.

The maximal stress in the primary foot ligaments is shown in Figure 7. Unlike in the joint cartilage, the maximal stress in almost all the primary foot ligaments increased with the material hardness and arch height of the orthopedic insoles.

## 4. Discussion

Correction and protection of orthopedic insole is a widely used physical therapy for the treatment of adolescent flat feet [16, 21, 22]. However, the corrective effectivity is significantly related with the insole shapes and material hardness. An optimal design of personalized orthopedic insole could greatly improve the foot supporting function and prevent the occurrence of symptomatic complications. Previous study showed that the custom-molded insole reduced maximum stress 40% more than the flat surface insole. In the increase of insole thickness, stress distribution becomes more uniform and maximum stress value decreases up to 10% [6].

It was reported that orthopedic insole could improve the patient's foot arch, hereby relieving foot pain, preventing inflammation of soft tissue and tendon sheath and other pathological features [7]. However, there is little knowledge about the changes in the internal skeleton and of medial longitudinal arch by wearing orthopedic insole. In this study, the influence of design parameters, including material hardness and support arch height of personalized orthopedic insole on the correction of foot arch and plantar pressure distribution, and the biomechanical effects on the foot tissues, including stress distribution on joint cartilage and ligaments, were quantitatively analyzed by in vivo experiment and finite element modeling. The results showed that the foot arch height increased with the material hardness and arch shape of the insole. The insole with harder material and higher scaffold of the medial longitudinal arch elevate higher foot arch [23–25]. By wearing the insole made up with the material of 40° and type III shape, the arch height was elevated up to twice of the initial bare foot weight-bearing condition.

Plantar pressure is one of the most important parameters reflecting the interaction between the foot and insole [5, 25, 26]. It was found that the hardness of the insole material will affect the plantar pressure distribution and the peak value, and affect the trajectory of plantar pressure center, and hereby affect trajectory of upper body gravity center [22]. Regardless the insole shape (types I, II, and III), the peak plantar pressure increased with the material hardness that means harder material results in higher plantar pressure. The peak plantar pressure was not apparently increased by wearing types I and II insoles, but apparently increased by wearing type III insoles. By wearing insoles made up by material with hardness of 40°, regardless the shapes (types I, II, and III), the plantar pressure was higher than the bare feet condition in which it was about 142 KPa.

Cartilage injury and joint dislocation are the primary reasons for foot pain in many patients [25]. Orthopedic insole is used to correct the foot arch height and relieve the pain. The purpose of correcting foot arch height is to redistribute the force transfer pattern in the joints to avoid further damaging of foot tissues. However, in the clinical practice, wearing orthopedic insoles is more painful than nonwearing condition in short-term follow-ups. It means that there is an effect on the foot tissues like the ligament and joints by wearing orthopedic insole, at last in short-term follow-ups. In the primary middle joints (talonavicular joint and calcaneocuboid joint), the maximal stress increased with the material hardness. In the calcaneocuboid joint, the stress increased with the arch height of insoles, meaning higher insole arch height results in greater joint stress. However, in the talonavicular joint, type II insole results in the greatest cartilage stress. Despite that, the stress in the middle joint was relatively much lower than that in the ankle joints (tibiotalar joint and talofibular joint), which are the primary structure transferring body weight to the foot. The material hardness did not apparently affect the stress in the ankle joints, but the arch height of orthopedic insoles did. However, the maximal stress in almost all the primary foot ligaments increased with the material hardness and arch height of the orthopedic insoles.

In a previous study, the authors found that by changing the material of the insole, the value of maximum stress remains nearly constant [6]. In this study, material hardness of 30°, 35°, and 40° and arch height of 27 mm, 30 mm, and 33 mm were chosen for analysis. The biomechanical effects on the flatfoot of the arch height (increase with 3 mm interval) were more sensitive than the material hardness (increase with 5 intervals). For example, the average plantar pressure with type I, type II, and type III insoles was 10.4, 14.0, and 16.4 MPa (standard deviation: SD = 3.0 MPa), respectively, while the average plantar pressure with material hardness of 30°, 35°, and 40° was 10.6, 14.3, and 15.8 MPa (SD = 2.7 MPa). The average stress in dorsal cuneonavicular ligament was 0.17 MPa, 0.32 MPa, and 0.52 MPa (SD = 0.17 MPa) for insole shape, but 0.32 MPa, 0.35 MPa, and 0.35 MPa (SD = 0.018 MPa) for material hardness.

Several limitations of this study should be noted for the interpretations and applications of the predicted results. The correction stage of flatfoot might influence the biomechanical evaluation eventually. However, the biomechanics was just evaluated for the first stage of orthopedic insole treatment. Evaluation of long-term effect should be done in the future. Since there were differences between individuals (degree of flatness, flexible of foot arch, material property, etc.), it is limited to apply the findings obtained from only one subject to all flatfoot. Fortunately, it was a self-comparison and parametric study, especially the FE foot model could represent patients with similar tissue geometry and arch height. Muscle forces play important role by keeping foot stability and providing stiffness. However, only tendon force was considered in the present study. The displacement of navicular bone based on the in vivo experiment was used as a displacement loading to simulate to corrective function of orthopedic insole, which might not reflect the interaction of insole and skin. In addition, the material property of the foot tissues which was simplified as linear formulation and achieved for the literature weakens the individual characters. However, as a parameterized study of material hardness and shape of insole, this simplification should induce a universal support of orthopedic insole design.

## 5. Conclusion

In general, insole material and support design are positively affecting the correction of orthopedic insole, but negatively resulting in unreasonable stress on the stress in the joint and ligaments. There should be an integration of improving correction and reducing stress in foot tissues.

## Figures and Tables

**Table 1:** Material mechanical properties and element types of the FE model.

Component	Element type	Young's modulus E (MPa)	Poisson's ratio	Cross-sectional area (mm^2^)
Bony structures	3D tetrahedra	7300	0.3	—
Soft tissue	3D tetrahedra	1.19	0.48	—
Plantar fascia	3D tetrahedra	350	0.35	290.7
Cartilage	3D tetrahedra	10	0.4	—
Ligaments	Tension-only truss	0~700	0.34	18.4~260
Skin	3D tetrahedra	1	0.4	—
Plantar support	3D hexahedron	21,0000	0.3	—
