# Corrigendum to “The Effect of Arch Height and Material Hardness of Personalized Insole on Correction and Tissues of Flatfoot”

**DOI:** 10.1155/2018/4136246

**Published:** 2018-01-16

**Authors:** Honglun Su, Zhongjun Mo, Junchao Guo, Yubo Fan

**Affiliations:** ^1^Key Laboratory for Biomechanics and Mechanobiology of Ministry of Education, School of Biological Science and Medical Engineering, Beihang University, Beijing 100086, China; ^2^Beijing Key Laboratory of Rehabilitation Technical Aids for Old-Age Disability, Key Laboratory of Rehabilitation Technical Aids Analysis and Identification of the Ministry of Civil Affairs, National Research Centre for Rehabilitation Technical Aids, Beijing 100176, China

In the article titled “The Effect of Arch Height and Material Hardness of Personalized Insole on Correction and Tissues of Flatfoot” [1], the name of the first author was given incorrectly as Shonglun Su. The author's name should have been written as Honglun Su. The revised authors' list is shown above.
